# Alternatively Spliced Isoforms of K_V_10.1 Potassium Channels Modulate Channel Properties and Can Activate Cyclin-dependent Kinase in *Xenopus* Oocytes*

**DOI:** 10.1074/jbc.M115.668749

**Published:** 2015-10-30

**Authors:** Fernanda Ramos Gomes, Vincenzo Romaniello, Araceli Sánchez, Claudia Weber, Pratibha Narayanan, Maryna Psol, Luis A. Pardo

**Affiliations:** From the ‡ Department of Molecular Biology of Neuronal Signals and; the § Oncophysiology Group, Max Planck Institute of Experimental Medicine, Hermann-Rein-Strasse 3, 37075 Göttingen, Germany

**Keywords:** cell cycle, glycosylation, post-transcriptional regulation, potassium channel, RNA splicing, KCNH1, K_V_10.1

## Abstract

K_V_10.1 is a voltage-gated potassium channel expressed selectively in the mammalian brain but also aberrantly in cancer cells. In this study we identified short splice variants of K_V_10.1 resulting from exon-skipping events (E65 and E70) in human brain and cancer cell lines. The presence of the variants was confirmed by Northern blot and RNase protection assays. Both variants completely lacked the transmembrane domains of the channel and produced cytoplasmic proteins without channel function. In a reconstituted system, both variants co-precipitated with the full-length channel and induced a robust down-regulation of K_V_10.1 current when co-expressed with the full-length form, but their effect was mechanistically different. E65 required a tetramerization domain and induced a reduction in the overall expression of full-length K_V_10.1, whereas E70 mainly affected its glycosylation pattern. E65 triggered the activation of cyclin-dependent kinases in *Xenopus laevis* oocytes, suggesting a role in cell cycle control. Our observations highlight the relevance of noncanonical functions for the oncogenicity of K_V_10.1, which need to be considered when ion channels are targeted for cancer therapy.

## Introduction

The human voltage-gated potassium channel, K_V_10.1 (*Ether-à-go-go*; *KCNH1*) is expressed physiologically in the brain, where it participates in the control of calcium entry into synapses during repetitive firing (1). It is not detectable in healthy tissues outside of the brain, but its aberrant expression in cancer cells has been reported frequently (2, 3). This ion channel is involved in the cell cycle progression of tumor cells, for which proliferation can be reduced by the inhibition of channel expression (4–6) or by treatment with classical channel blockers (7) or a functional monoclonal antibody (8). A mutation precluding potassium permeability does not completely abolish xenograft tumor formation by transfected cells (7), indicating that the contribution of K_V_10.1 to tumor progression does not fully rely on its primary function as an ion channel. The functional protein is a homo- or heterotetramer, where each monomer is comprises six transmembrane domains and large cytoplasmic N and C termini. Potentially relevant functional domains can be recognized on both intracellular ends; these are, most prominently, the calmodulin binding domains (9–11); a cyclic nucleotide binding domain (12–15); a Per-Arnt-Sim (PAS) domain in the N terminus (16, 17); a nuclear localization signal (NLS) (18); and a C-terminal tetramerizing coiled-coil (TCC)^5^ domain, which is relevant for tetrameric assembly (18–21). The function of the channel can be also modified by a number of different interacting proteins such as (besides calmodulin) potassium channel regulator 1 (KCR1), calmodulin kinase, 14-3-3, epsin, rabaptin 5, cortactin, or PIST (PDZ domain protein interacting specifically with TC10) (22–28). Mature K_V_10.1 channels undergo glycosylation on asparagines 388 (only core-glycosylated) and 406 in the extracellular loop between the S5 and S6 transmembrane domains (29), shifting the apparent molecular mass from 100 to 110 and 130 kDa, respectively, as assessed by Western blot.

Alternative splicing is a common post-transcriptional modification leading to expanded proteomic complexity. 95% of multi-exon human genes exhibit alternative splicing (30), and so do ion channels. Alternative splicing resulting in changes in the pharmacological profile, electrophysiological properties, surface expression, or intracellular localization of ion channels has been described in other channels (31–43). In some cases, the resulting splice variant lacks conductive properties and acts as a dominant negative (44–46).

Two splice variants of the mammalian K_V_10.1 have been identified, termed K_V_10.1a and K_V_10.1b (4, 47, 48). Both form active ion channels with properties that are very similar to each other. A third splice variant cloned from *Drosophila* (Eag80) is composed of only the N and C termini of the channel. Although it does not produce an active ion channel, it activates a signaling cascade leading to altered cell architecture (49). The aim of the present work was to test the occurrence and to study the biological relevance of alternative splicing in human K_V_10.1 channels. The knowledge about the contribution of noncanonical ion channel splice variants is crucial to understanding the mechanisms underlying the progression and resistance of oncologic diseases.

## Experimental Procedures

### 

#### 

##### Cells

Cell lines DU145 (ACC 261), HEK293 (ACC 305), HeLa (ACC 57), IPC298 (ACC 251), IGR39 (ACC 239), IMR32 (ACC 165), and SH-SY5Y (ACC 209) were purchased from DSMZ (Braunschweig, Germany). MDA-MB-435S (HTB129) cells were from ATCC (Manassas, VA), PNT2 cells (ECACC95012613) were obtained from ECACC (Salisbury, UK). GL15 cells were kindly provided by Dr. Fioretti (University of Perugia, Italy). Each cell line was cultured in their respective recommended medium supplemented with 10% FCS (PAA Laboratories) at 37 °C in humidified 5% CO_2_ atmosphere. For stablly transfected cell lines (HEK expressing K_V_10.1 in the pTracerCMV vector, a cell line routinely used in our laboratory (4, 6–8), the selection compound Zeocin (Calya) was added to the culture medium at 3 μg/ml. Transient transfections were performed using FuGENE (Roche Applied Science) or Lipofectamine 2000 (Invitrogen). Proliferation was estimated using Alamar Blue (BIOSOURCE) or WST assays (Roche Applied Science) as described (50) or by live cell imaging in an IncuCyte Zoom system (Essen Biosciences) to determine the percent confluence as a function of time.

##### Molecular Biology

The splice variants were cloned in expression vectors by substituting the 1834-bp AarI-BspEI fragment from the relevant plasmids containing K_V_10.1 (AarI cuts at exon 3 and BspEI at exon 11) with the corresponding fragment from the PCR amplicons cloned in pGEM-T (310 bp for E65 and 763 bp for E70). The host vectors were pSGEM-K_V_10.1 for efficient expression in the *Xenopus* system (51) and pcDNA3-K_V_10.1 and pcDNA3-K_V_10.1-mVenus (18) for expression in mammalian cell lines and generation of the mVenus fusions, respectively, where the fluorescent reporter is fused to the C-terminal end of the protein.

Site-directed mutagenesis was performed using QuikChange II XL site-directed mutagenesis kit (Agilent Technologies) according to the manufacturer's instructions. The primers for the E65^L405Y^ and E70^L556Y^ mutations were: 5′-AGGAGGACATCAAGGCCTACAACGCCAAAATGACCAATA-3′ and 5′-TATTGGTCATTTTGGCGTTGTAGGCCTTGATGTCCTCCT-3′. All constructs and amplicons were verified by sequencing.

Total RNA was obtained from cell pellets using an RNeasy mini kit (Qiagen), and 2.5 μg of RNA was used for cDNA synthesis using a SuperScript first-strand synthesis kit (Invitrogen). RNA and DNA concentration and yield were determined by optical density measurements at 260 and 280 nm using a spectrophotometer (UV-visible NanoPhotometer, Implen). Total and Poly(A)^+^ RNA from human brain (hBrain) was purchased from Clontech. The T7 mMessage mMachine kit (Ambion) using the T7 promoter from the pSGEM vector was used to prepare cRNA.

K_V_10.1 siRNA (target sequence: 5′-TACAGCCATCTTGGTCCCTTA-3′) was designed with the HiPerformance siRNADesign algorithm (BIOPREDsi). siRNAs (30 nm) were transfected using DreamFect (Oz Biosciences) or nucleofection (Lonza) for electrophysiological experiments (Solution L, programs T-020 for IPC298 and T-030 for IGR39). The negative control was the reverse but noncomplementary (“scrambled”) sequence of K_V_10.1.

Nested PCR was performed using 1.5 μl of first-strand cDNA in a 25-μl reaction volume for 20 cycles (95 °C for 30 s, 59 °C for 30 s, and 72 °C for 3 min with an initial denaturation step of 3 min at 95 °C). 1 μl of the reaction was used as the template for the second round (35 cycles). The amplification products were analyzed by electrophoretic separation in agarose gel followed by cloning in pGEM-T vector and sequencing in all cases.

5′-RACE was carried out using the 5′/3′-RACE kit from Roche Applied Science. 2 μg of total RNA extracted from melanoma cell lines or commercial human brain RNA (Clontech) was used for cDNA synthesis using external primers 2 and 1, respectively (Table 1). The dA-tailed cDNA was amplified at 52 °C using the oligo(dT) anchor primer and the specific primer used to synthesize the cDNA. Nested PCR was required to generate a visible product. 1.5 μl of the first round PCR reaction was amplified at 52 °C using the oligo(dT) anchor and the primers Internal 1 for the melanoma cell lines and Internal 2 for human brain. The PCR products were gel-purified and cloned into pGEM-T Easy vectors (Promega) for sequence analysis. The 3′-UTR region was amplified using nested PCR as described above and sequenced. The primers are listed in Table 1.

**TABLE 1:** **Sequences of primers used**

	Primers	Sequence
Nested PCR	External forward	5′-TGT TCG GCG GTC CAA TGA TAC TAA-3′
	External reverse	5′-TCC CGG CCC CCT CTC TCA-3′
3′-UTR	External forward	5′-ACC GTG CGT GAG AGT CCT-3′
	External reverse	5′-TTC TCG GCA CTT TCC CAC C-3′
	Internal forward	5′-GGC AGC CTC CAC CTC CG-3′
	Internal reverse	5′-ATG GCT GCT GCT CTG TTC TG-3′
5′-RACE	External 1	5′-TGC AGATCA CAG TAG GTC AAG GC-3′
	External 2	5′-AAT CCA TTA CTC GCT CAC TCA-3′
	Internal 1	5′-TTC AAT CGT GTC TTT ATC AG-3′

##### Ribonuclease Protection Assay

Ribonuclease protection assays were carried out using the RPA III kit (Ambion) according to the manufacturer's instructions. Briefly, a K_V_10.1 cDNA fragment (342 bp for E65 and 197 bp for E70) encompassing the specific exon junctions for each variant was generated by PCR from plasmid DNA and subcloned into pSGEM, and the plasmid was used as a template to synthesize ^32^P-labeled RNA probe (MAXIscript kit, Ambion). 200,000 cpm of purified ^32^P-labeled RNA was hybridized overnight at 42 °C with 3 μg of mRNA isolated from confluent tumor cells (using the Micro-FastTrack 2.0 kit, Invitrogen). Digestion was then performed for 30 min at 37 °C with an RNase A/RNase T1 mix (Ambion). 100 μg of yeast total RNA was used as a negative control to test for the presence of probe self-protection bands. The samples were run on a 5% polyacrylamide gel and exposed for 1–4 days.

##### Northern Blot

For separation of mRNA, a 2% formaldehyde MOPS-agarose gel was used and left running for 7 h; the bands were transferred to a Hybond N membrane overnight and cross-linked (UV Stratalinker 1800, 1200 J, Stratagene). The dry membrane was washed with 2× SSC for 10 min, prehybridized with Rapid-hyb buffer (Roche Applied Science) for 2–3 h, and incubated overnight at 65 °C with a 700-bp C-terminal [^32^P]dCTP-labeled K_V_10.1 fragment in Rapid-hyb buffer plus. The probe was generated using a DecaLabel DNA labeling kit (Fermentas) and purified with Illustra MicroSpin G-50 columns (GE Healthcare). After overnight hybridization the membrane was collected and washed twice (30 min each) with 2× SSC and again twice with 2× SSC + 0.1% SDS at 65 °C. The membrane was then put in contact with an x-ray film and exposed for 4 h or overnight.

##### Immunofluorescence

Rabbit polyclonal anti-K_V_10.1 antibodies were generated in our laboratory and have been described elsewhere (2). The polyclonal antibody 9391 and mAb33 recognize the C terminus of K_V_10.1, whereas mAb62 binds to the extracellular S5-S6 linker. ToPro3 (Invitrogen) was used to label the nuclei. Briefly, cells were washed three times with TBS (150 mm NaCl and 20 mm Tris-HCl, pH 7.5), fixed with 4% *p*-formaldehyde (4 °C for 4 min), and permeabilized with 1% Triton X-100 in TBS for 10 min. Nonspecific binding was blocked with 10% bovine serum albumin in TBS for 30 min. Primary antibody (1 μg/ml) incubation was done at room temperature for 2 h. Alexa Fluor 546-labeled anti-mouse IgG antibody (Molecular Probes) was used as a secondary antibody. The coverslips were mounted using ProLong (Molecular Probes) and observed under a Zeiss LSM 510 Meta laser-scanning confocal microscope.

##### Protein Extraction, Separation, and Western Blot

To obtain cell lysates, cultures were washed twice with phosphate-buffered saline, incubated in 3 ml of lysis buffer (mm: 50 Tris-HCl, pH 7.4, 300 NaCl, 5 EDTA, and 1% Triton X-100 containing protease inhibitor mixture (Roche Applied Science)) for 30 min, and centrifuged for 15 min at 21,300 × *g*. The supernatant was used as the total cell extract. Protein concentration was determined using BCA protein assay reagent (Pierce). To obtain oocyte lysates, 15 oocytes were incubated in 300 μl of oocyte lysis buffer (1% Triton X-100, 150 mm NaCl, 20 mm Tris-HCl, 5 mm MgCl, and 5 mm EDTA) for 30 min and centrifuged twice for 2 min at 21,300 × *g*.

Immunoprecipitations were performed using protein G-magnetic beads (New England Biolabs). 1 mg of protein extract was incubated with the relevant antibody: mAb33 (3 μg, overnight incubation) or mAb62 and anti-*Xenopus* cyclin B2 (Santa Cruz Biotechnology).

Proteins were separated by SDS-PAGE (NuPAGE Novex Tris acetate 3–8% gel, Invitrogen) and transferred to nitrocellulose membranes (GE Healthcare). The membranes were probed with polyclonal 9391 anti-K_V_10.1 (dilution 1/1500) or 6551 anti-GFP (dilution 1/1000, Abcam) and developed using the Millipore Immobilon system. Signals were detected in a Bio-Rad ChemiDoc luminescence detection system.

##### Glycosidase Digestion

200 μg of protein lysate was immunoprecipitated as described above. The magnetic beads containing the immunoprecipitated proteins were resuspended in 2 μl of elution solution (100 mm β-mercaptoethanol in 0.1% SDS). Samples were then heated up to 99 °C for 10 min, centrifuged briefly, and exposed to a magnetic field. At this point, the 21 μl of supernatant from each sample was distributed in three microcentrifuge tubes for treatment with 1 μl of Endo H (5 units/ml, Sigma), 1 μl of PNGase F (≥2 units/ml, Sigma), and a control tube containing immunoprecipitated samples that were not treated with enzyme. The tubes were incubated overnight at 37 °C, and then the protein was separated by SDS-PAGE as described above.

##### Cdc2 Kinase Activity

Oocytes extracts were incubated in a solution containing biotinylated synthetic peptide (Ser-Leu-Tyr-Ser-Ser-Ser-Pro.Gly-Gly-Ala-Tyr-Cys) peptide for 30 min at 30 °C (Cdc2 substrate; MESACUP Cdc2 kinase assay kit, MBL International Corp.). The mixture was then transferred to a microwell plate coated with anti-phospho-MV peptide monoclonal antibody (4A4). The retained biotinylated phospho-MV peptides were subsequently detected with streptavidin-HRP and ABTS (Invitrogen) measured in a plate reader at 405 nm (reference 490 nm).

##### Surface Labeling

Cells expressing K_V_10.1-BBS (52) were incubated for 10 min on ice with 2.5 μg/ml α-bungarotoxin (BTX)-biotin (Invitrogen), washed twice with ice-cold PBS, scraped, and centrifuged at 800 × *g* for 3 min. Cell pellets were resuspended in 20 mm Tris-HCl, 150 mm NaCl, 5 mm MgCl_2_, and 1% Nonidet P-40, pH 7.4, containing protease inhibitors. The lysates were passed several times through a 25-gauge needle, placed in microcentrifuge tubes, incubated for 20 min on ice, and finally centrifuged at 16,100 × *g* for 15 min at 4 °C. Streptavidin-coated plates (Thermo Scientific) were washed twice with PBS containing 0.05% Tween 20, 0.1% BSA, and 0.1% Triton X-100. 30 and 150 μg of protein (in triplicates) were then incubated for 30 min on ice. The plates were washed twice and then blocked for 30 min with NPE (150 mm NaCl, 5 mm EDTA, 50 mm Tris, 5 mm KCl, and 1% Nonidet P-40, pH 7.5) containing 1% casein. Anti-K_V_10.1 antibody (mAb62) was diluted in NPE with 0.1% casein and used at 2.5 μg/ml for 90 min. The wells were washed three times with NPE with 0.1% casein and blocked again in NPE with 1% casein for 30 min. Secondary antibody (1:500 ECL HRP-linked anti-mouse IgG (GE Healthcare)) was then incubated for 90 min. After seven washes with NPE-0.1% casein, peroxidase activity was determined using ABTS as described above.

##### Patch Clamp

Recordings were performed in the whole-cell configuration using an EPC9 amplifier and Pulse software (HEKA, Lambrecht, Germany). Currents were filtered at 10 kHz and digitized at 50 kHz. Patch pipettes were pulled from Corning No. 0010 glass (World Precision Instruments) to resistances of 2–3 megohms. Solutions for HEK293-K_V_10.1, IPC298, and IGR39 melanoma cells contained the following compounds (in mm): internal, 100 KCl, 45 *N*-methyl-d-glucamine, 5 1,1-bis(*O*-aminophenoxy)ethane-*N*,*N*,*N*,*N*-tetracetic acid (BAPTA), 5 EGTA, 1 MgCl_2_, and 10 HEPES, pH 7.4; external, 160 NaCl, 2.5 KCl, 2 CaCl_2_, 1 MgCl_2_, 8 glucose, and 10 HEPES, pH 7.4. Series resistance was compensated to ∼85%. To determine the K_V_10.1 current amplitude, we applied a conditioning pulse to −100 mV for 1500 ms to slow down the activation of hEag1, and the outward currents were then elicited by a square depolarization to +40 mV for 500 ms. The *I*-V protocol consisted of 250-ms (or 500-ms) voltage pulses ranging from +80 mV to −60mV in 20-mV decrements. To compare voltage-elicited current response between different groups of cells, the current was first normalized to the cell size (as measured by C_slow_). The average current density in the segment between 80 and 90% of the duration of the stimulus 80 to 90% time of each pulse and plotted against the voltage.

Nonstationary noise analysis was carried out in the outside-out configuration on macropatches of *Xenopus* oocytes injected with the relevant cRNAs (53). This preparation was necessary to have enough current in a membrane patch. Patches were obtained from the membrane of oocytes devoid of vitelline membrane using 0.9–2-megohm pipettes filled with 100 mm KCl, 10 mm EGTA, and 10 mm HEPES, pH 7.2. Currents were sampled at 20 kHz and filtered at 4 kHz. Variance/current plots were constructed using PulseTools software (HEKA) from several hundred consecutive 50-ms depolarizations to +40 mV from a holding potential of −80 mV. The number of available channels (*n*) and the single-channel current (*i*) were determined from the variance *versus* average macroscopic current (*I*) plot using the equation

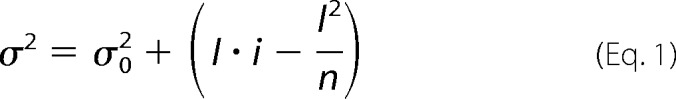
 where σ__0__^2^ represents the baseline variance.

##### Two-electrode Voltage Clamp

Oocytes were injected with 50 nl of a 1 ng/μl cRNA solution and kept at 18 °C in ND96 solution (96 mm NaCl, 2 mm KCl, 0.2 mm CaCl_2_, 2 mm MgCl_2_, 0.5 mm theophylline, 0.1 mm gentamycin, and 5 mm HEPES, pH 7.5). Currents were recorded 1–3 days after cRNA injection, using a Turbo TEC-10CD amplifier (NPI Electronic Instruments) at room temperature. The intracellular electrodes had resistances of 0.3–1.5 megohms when filled with 2 m KCl. The extracellular measuring solution contained (mm): 115 NaCl, 2.5 KCl, 1.8 CaCl_2_, and 10 HEPES/NaOH, pH 7.2. Data acquisition and analysis were performed with the Pulse/PulseFit (HEKA Electronics) and IgorPro (WaveMetrics) software packages. Current records were filtered at 1 kHz. The membrane potential was held at −80 mV. To characterize current-voltage relationships, an *I*-V protocol was used, generally consisting of 250-ms voltage pulses ranging from +80 mV to −60 mV in 20-mV decrements. To compare voltage-elicited current responses between different groups of oocytes, the average steady state current between 80 and 95% of the duration of the pulse was plotted against the voltage.

##### Statistics

Data are presented as mean ± S.E. for the indicated number of experiments. The methods for determining statistical significance are indicated in the corresponding figure legends.

## Results

### 

#### 

##### K_V_10.1 Variants in Melanoma Cell Lines

**FIGURE 1.**
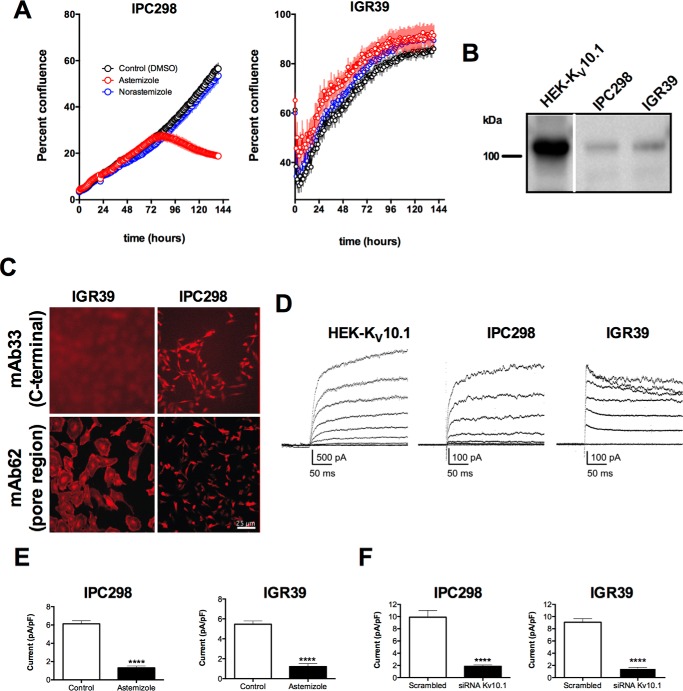
**K_V_10.1 protein detection in melanoma cell lines.** *A*, astemizole (*red symbols*) reduces proliferation (measured as percent confluence) in melanoma cell lines as compared with norastemizole (*blue symbols*) and vehicle control (*black symbols*). Both drugs were used at 500 nm. In IPC298 cells the proliferation was strongly reduced by astemizole treatment, whereas in IGR39 cells there was no effect. *B*, the cell extracts were precipitated using a mixture of two anti-K_V_10.1 monoclonal antibodies (mAb33 against the C terminus and mAb62 against the pore region of the channel) and detected with an anti-K_V_10.1 polyclonal antibody. *C*, K_V_10.1 immunostaining using mAb33 (*upper panel*) and mAb62 (*lower panel*) for IGR39 (*left*) and IPC298 (*right*) melanoma cell lines. *D*, K_V_10.1 currents elicited in HEK293-K_V_10.1 (*left traces*), IPC298 (*middle traces*), and IGR39 (*right traces*) cell lines. HEK293-K_V_10.1 (*n* = 21), IPC298 (*n* = 82), and IGR39 (*n* = 121). *E*, reduction on K_V_10.1 currents by 5 μm astemizole. Current amplitudes were determined as mean current in the last 100 ms of a 1.3-s depolarization to +60 mV for 9 (IPC298, *left*) and 10 cells (IGR39, *right*) *F*, reduction of K_V_10.1 currents by a K_V_10.1 siRNA. Cells were transfected with siRNA for K_V_10.1 and currents measured after 30 h, as described for *E*. The graphs for IPC298 (*left panel*) (scrambled *n* = 8, siRNA *n* = 11) and IGR39 (*right panel*) (scrambled *n* = 9, siRNA *n* = 10) show that more than 80% of the current was blocked by the siRNA against K_V_10.1. ****, *p* < 0.0001.

Astemizole reduces the proliferation of many cancer cell lines, and its effect can be at least in part attributed to inhibition of the K_V_10.1 channel (7, 54). We sought to compare the effects of this drug in two different melanoma cell lines, IPC298 and IGR39. As controls for these experiments we used dimethyl sulfoxide and norastemizole, a metabolite of astemizole that does not block K_V_10.1. We observed an intense effect of astemizole on the proliferation of IPC298 but no effect on IGR39 cells (Fig. 1*A*). This was in agreement with previous reports that IGR39 shows little or no K_V_10.1 expression (55); however, in our hands both cell lines were K_V_10.1-positive (see Table 2 and Ref. 6). To clarify this discrepancy, we tested whether the channel protein was present in both cell lines. First, we performed immunoprecipitation with a mixture containing two monoclonal antibodies: mAb33 (its epitope maps to the C-terminal end) and mAb62 (directed against the pore region). The presence of K_V_10.1 in the immunoprecipitate was subsequently tested by Western blot analysis using a polyclonal, C-terminal-directed antibody. A band of the expected size was detected in both cell lines, indicating that K_V_10.1 is expressed in both IPC298 and IGR39 cells (Fig. 1*B*).

**TABLE 2:** **K_V_10.1 expression in human brain and melanoma cell lines (assessed by real-time PCR)** hTfR, human transferrin receptor.

Cell line	K_V_10.1 expression normalized to hTfR	Standard deviation
Human brain	1.195	0.097
Melanoma IGR39	0.058	0.007
Melanoma IPC298	0.031	0.002

We also stained both cell lines with the two mentioned monoclonal anti-K_V_10.1 antibodies (Fig. 1*C*). Both antibodies labeled IPC298 cells with similar efficiency and gave rise to the same patterns. In IGR39 cells, staining with the pore antibody resulted in a clear, peripherally distributed signal (Fig. 1*C*), but the antibody against the C terminus of K_V_10.1 did not produce any signal. The polyclonal C-terminal antibody used for Western blot experiments was generated against the whole C terminus of the channel, whereas the epitope for the monoclonal counterpart corresponded to the TCC domain. Therefore, the lack of signal using the monoclonal antibody indicates that the epitope is either masked or absent in these cells.

##### Electrophysiology of IPC298 and IGR39 Melanoma Cell Lines

To test whether the expression of K_V_10.1 protein has a functional correlate, we studied the electrophysiological properties of the IPC298 and IGR39 cell lines by using whole-cell patch clamp. Representative current traces from both cell lines are shown in Fig. 1*D*. In IPC298 cells (Fig. 1*D*, *middle traces*), depolarization induced a slowly activating/non-inactivating outward current with kinetics compatible with K_V_10.1. For comparison, typical K_V_10.1 traces from transfected HEK-K_V_10.1 cells are also shown (Fig. 1*D*, *left traces*). In contrast, IGR39 cells showed a current with faster activation and partial inactivation (Fig. 1*D*, *right traces*). This current, kinetically very different from K_V_10.1, was blocked by imipramine (not shown) and astemizole (Fig. 1*E*) at concentrations reported to block K_V_10.1 (56). Most significantly, the current was abolished by treatment with K_V_10.1-specific siRNA (6) (Fig. 1*F*), strongly suggesting that the detected current requires the presence of K_V_10.1 mRNA. The siRNA recognizes nucleotides 690–710 of the open reading frame, corresponding to exon 6, which encodes the transmembrane core of the protein. These results suggested the possibility that the differential electrophysiology is related to the presence of different splice variants in the IPC298 and IGR39 cell lines.

##### K_V_10.1 Splice Variants in Cancer Cells and Brain Tissue

**FIGURE 2.**
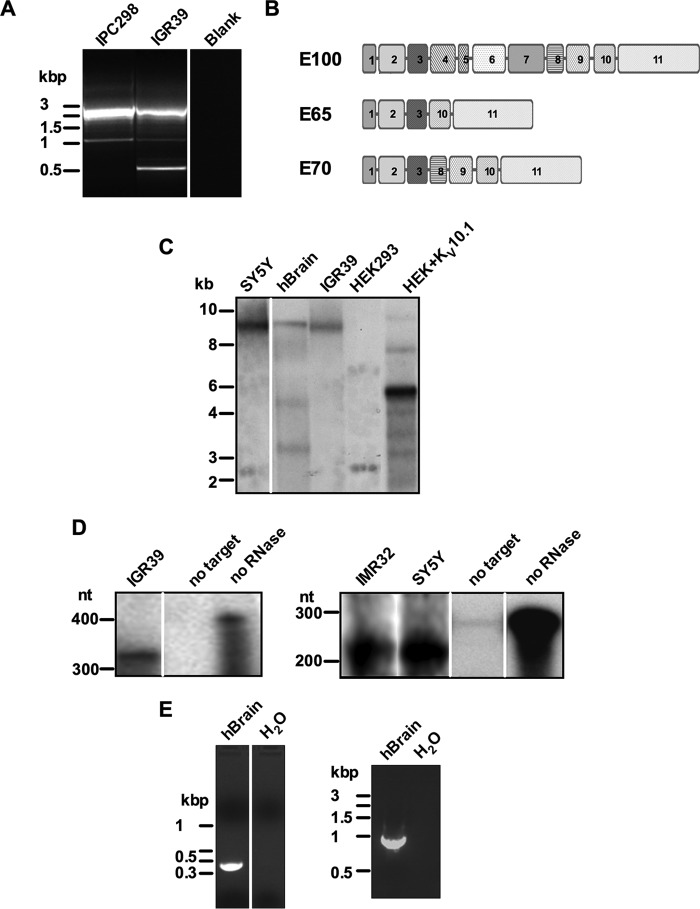
**Detection of K_V_10.1 splice variants in human cell lines and brain tissue.** *A*, 1.5% agarose gel with nested PCR products obtained with the specific primers for K_V_10.1 listed in Table 1. *Lanes 1* and *2*, cDNAs for IPC298 and IGR39 cells, respectively; *lane 3*, blank. *B*, scheme of the exon structure of K_V_10.1 (E100) and the splice variants E65 and E70. *C*, Northern blot analysis of variant mRNA expression in human tissues and human cells. 30 μg of mRNA from the cell lines and tissues indicated was separated in a 2% formaldehyde agarose gel, transferred to Hybond N membranes, and hybridized overnight to a 700-bp DNA probe for the C terminus of K_V_10.1 channel. *D*, detection of E65 and E70 splice variant by ribonuclease protection assay. Two antisense ^32^P-labeled RNA probes (342 bp for E65 and 197 bp for E70) were hybridized with mRNA isolated from tumor cells. The probes protected from RNase digestions were analyzed by polyacrylamide gel electrophoresis and autoradiography. The 342-bp (*left panel*) and the 197-bp (*right panel*) protected fragment represent the 3–10 and the 3–8 exon junctions, respectively. *E*, 5′-RACE (*left panel*) and 3′-UTR amplification (*right panel*) in human brain tissue. The amplified products from RACE amplifications and 3′-UTR nested PCR were separated in 1,5% agarose gel.

To screen for candidate K_V_10.1 variants, we performed nested PCR on cDNA synthesized using oligo(dT) with primers located on exons 2 and 11 (Table 1). The predicted PCR product for the full-length channel referred to here as E100, after the predicted protein size) was ∼1.9 kbp. In addition to this product, a prominent band of ∼0.9 kbp was detected in IPC298 cells and another band of 0.5 kbp in IGR39 cells, although the latter cell line also shows the 0.9-kbp band with lesser abundance (Fig. 2*A*). Cloning and sequencing of the amplicons showed the expected K_V_10.1 sequence for E100. In the case of IGR39 cells, analysis of the 0.5-kbp fragment revealed a sequence that stopped at the end of exon 3, skipped exons 4 to 9, and continued at the beginning of exon 10. This would give rise to a protein of ∼65 kDa, which we will hereafter refer to as E65. Sequence analysis of the 0.9-kbp fragments revealed that the end of exon 3 was joined with the beginning of exon 8 from K_V_10.l. Following the same logic as for E65, we will term this variant E70 (Fig. 2*B*). In both E65 and E70 the reading frames were conserved, and no new stop codons were generated. The skipped exons encode for the transmembrane domains of the ion channel; translation of this shorter mRNA is expected to produce non-channel proteins, composed only of the N and C termini of K_V_10.1. Nested PCR was also performed using additional K_V_10.1-expressing cancer cell lines as well as normal human brain. E65 was found in DU145, PNT2, and SH-SY5Y cells. E70 was amplified in SH-SY5Y and IMR32 cells and human brain. GL15, HeLa, MCF7, and MDA-MB-435S cells were negative for both splice forms.

The presence of shorter K_V_10.1 splice variants was confirmed by Northern blot. Purified mRNAs from human brain and SH-SY5Y and IGR39 cells (30 μg) were hybridized to a labeled probe targeting the K_V_10.1 C-terminal region (corresponding to exons 9–11), expected to be shared by all splice isoforms. This probe hybridized with three RNA bands on brain samples: one migrating at ∼9kb and two others having electrophoretic mobility of ∼5 and ∼3.5 kb. The ∼9-kb band is compatible with full-length K_V_10.1 transcript (4, 57) and was also detected in SH-SY5Y and IGR39 cells (Fig. 2*C*). All detected alternative splicing forms were expressed at low levels.

We then sought to confirm the presence of E65 and E70 using a ribonuclease protection assay. Purified mRNAs isolated from diverse K_V_10.1-expressing cancer cell lines were hybridized with radiolabeled antisense probes specifically targeting E65 and E70 transcripts (human brain RNA was not included in this approach for availability reasons). Single-stranded unprotected mRNAs were digested, and the resulting products were separated on a polyacrylamide gel. A 342-nt band, corresponding to the length of the E65 probe, was detected in IGR39 cells. E70 was detected in IMR32 and SH-SY5Y cell extracts (Fig. 2*D*). These experiments demonstrated the expression of E65 and E70 transcripts in native systems.

To exclude the presence of alternatively spliced regions or sequence differences in the 5′- and 3′-untranslated ends of the mRNA, 5′-RACE PCR was carried out. Primers located in regions common to all variants (Table 1) were used, and a unique band of 350 bp (Fig. 2*E*, *left panel*) was amplified, the sequence of which was identical to the expected K_V_10.1 sequence. 3′-RACE using either conventional or thermostable polymerases was systematically unsuccessful, arguably because of the abundance of GC-rich sequences (58) in that region of K_V_10.1. Nested PCR using primers placed at exon 11 and at the 3′-UTR to amplify the 3′-UTR boundaries (Table 1) produced a single 900-bp band (Fig. 2*E*, *right panel*). Sequencing of the amplicon did not reveal divergences from the expected K_V_10.1 sequence. In summary, we found no evidence of the presence of alternatively spliced regions at the 5′- and 3′-ends of IGR39 cells (not shown) and human brain (Fig. 2).

##### Physical Interaction of E65 and E70 with K_V_10.1

In numerous ion channels, splice variants interact with the corresponding full-length channel, modulating its function (37, 39, 46, 59). To test whether K_V_10.1 interacts with E65 and/or E70, we performed co-immunoprecipitation (co-IP) using extracts from HEK cells stably expressing K_V_10.1 (7, 56) and co-transfected with E65 or E70 constructs tagged with the fluorescent red-shifted GFP-derivative monomeric Venus (mVenus; expected molecular mass of the fusion proteins was 80 and 95 KDa, respectively). When E65mVenus and E70mVenus were immunoprecipitated using an anti-GFP antibody, Western blotting with an antibody against the C terminus of K_V_10.1 (therefore recognizing all isoforms) revealed the presence of a band compatible with full-length K_V_10.1. Co-IP with E65mVenus was more efficient than E70mVenus, but both splice variants were able to pull down the full-length protein.

**FIGURE 3.**
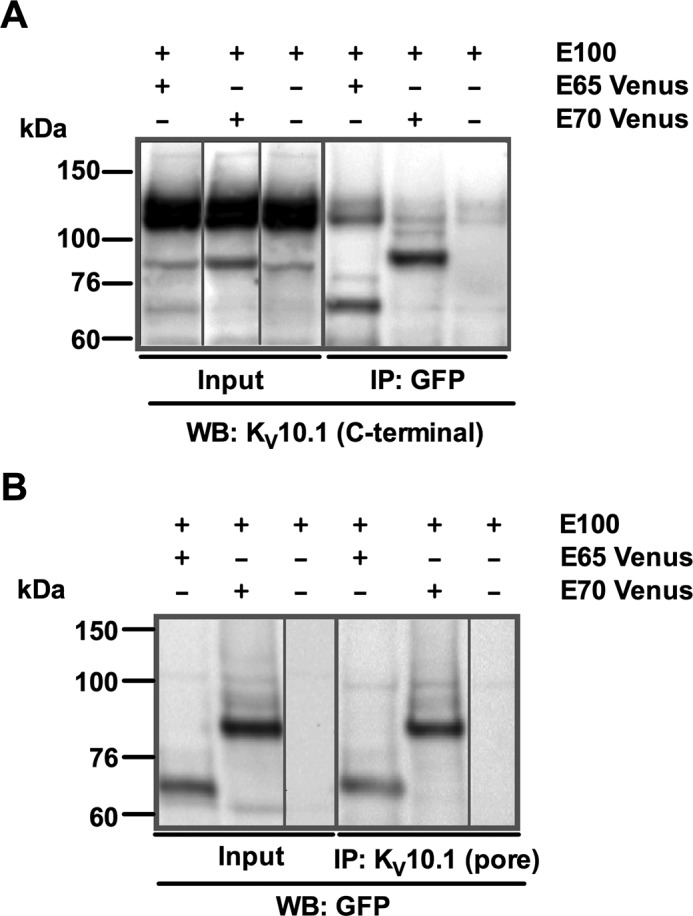
**E65 and E70 co-precipitate with E100.** *A*, *right panel*, immunoprecipitation of mVenus isoforms with anti-GFP antibody co-precipitated E100 protein of ∼130 and ∼110 kDa in E100 + E65 samples and E100 protein of ∼110 and ∼100 kDa in E100 + E70 samples. *Left panel*, input samples consisting of 10% (20 μg) not immunoprecipitated cell extracts. *B*, *right panel*, immunoprecipitation of E100 with a K_V_10.1 transmembrane domain-targeting antibody co-precipitated E65 and E70 proteins of ∼65 and ∼70 kDa in E100 + E65 and E100 + E70 samples, respectively. *Left panel*, input samples consisting of 10% (20 μg) non-immunoprecipitated cell extracts. As a detection antibody anti-GFP was used in both the *left* and the *right panels. WB*, Western blot.

The bands detected were qualitatively different. Whereas E65mVenus pulled a diffuse band similar to the majoritarian form present in the inputs, E70mVenus immunoprecipitates showed two sharper bands of smaller size (Fig. 3*A*, *right panel* (*IP: GFP*)), compatible with the partially (110 kDa) and fully (100 kDa) deglycosylated forms of the channel, respectively.

The reverse co-IP using a monoclonal anti-K_V_10.1 antibody directed against the pore region (which therefore does not bind to any of the shorter variants) developed with anti-GFP antibody revealed the presence of E65mVenus or E70mVenus in the corresponding extract, thus confirming the interaction (Fig. 3*B*). We concluded that both E65 and E70 interact with the full-length channel in living cells.

##### E65 and E70 Interaction with K_V_10.1 Alters the Abundance and Glycosylation Pattern of the Full-length Channel

**FIGURE 4.**
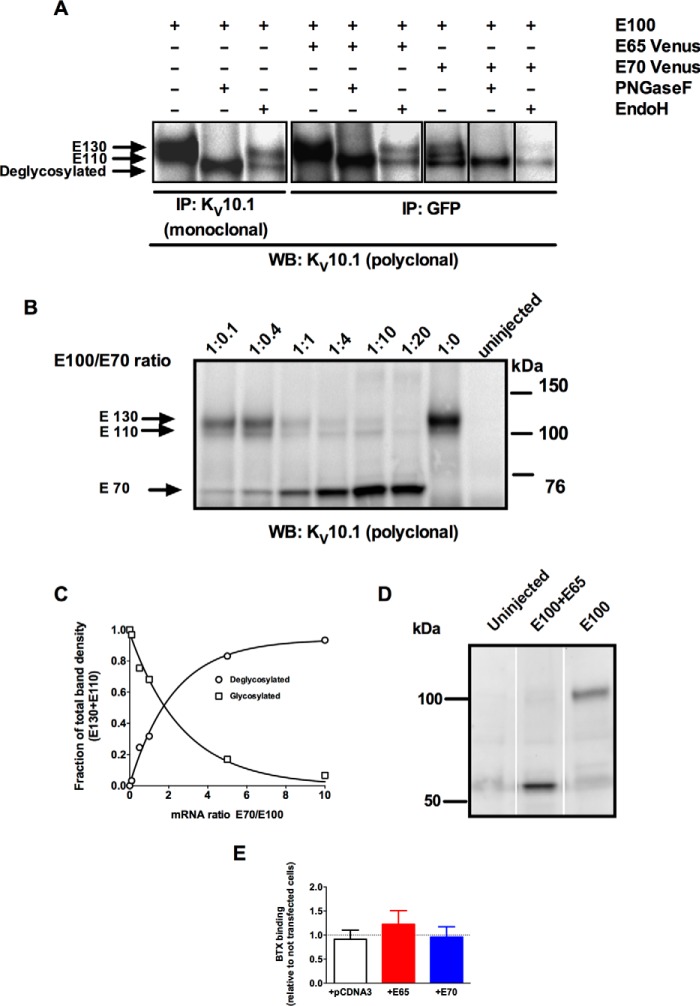
**Analysis of E100 glycosylation upon co-expression with E65 and E70.** *A*, co-immunoprecipitation with an anti-K_V_10.1 monoclonal antibody (mAb33) of E100 with the short isoforms E65 and E70 was performed, and precipitates were digested using Endo H and PNGase F to highlight the E100 glycosylation pattern. Notice that E130 is virtually absent when E100 is co-expressed with E70. *B*, immunoprecipitation of E100 and E70 proteins in oocytes injected with increasing ratios of E70/E100. The E110/E130 ratio increases together with increasing ratios of E70/full-length channel cRNA injected. *C*, densitometry analysis of data presented in Fig. *B. D*, immunoprecipitation of E100 with the short isoform E65 performed in oocyte lysates (15) using an anti-K_V_10.1 monoclonal antibody (mAb33) and detected with an anti- K_V_10.1 polyclonal antibody. Notice that E100 is strongly reduced when co-expressed with E65 at a 1:10 ratio. *E*, surface expression of the full-length form is not reduced by the spliced variants. Cells expressing a K_V_10.1 carrying an extracellular BTX binding site were labeled with biotinylated BTX, and the amount of label incorporated was quantified by sandwich ELISA (*n* = 9). *WB*, Western blot.

The typical K_V_10.1 glycosylation pattern (after immunoblotting using a specific antibody) consists of two distinct bands with electrophoretic mobility corresponding to ∼110 and ∼130 kDa (E110 and E130). E110 corresponds to core glycosylated K_V_10.1 located in the endoplasmic reticulum and the Golgi, whereas E130 is a post-endoplasmic reticulum complex glycosylated species (29). If K_V_10.1 is completely deglycosylated by PNGase F, the apparent size of the gel is ∼100 kDa. Co-IP showed different band patterns of full-length K_V_10.1 depending on which of the isoforms was co-expressed, suggesting differences in glycosylation (Fig. 4*A*). To characterize the glycosylation status of the immunoprecipitated bands we used Endo H (which removes only core oligosaccharides) or PNGase F (which fully deglycosylates proteins). The protein pulled down by E65 showed a pattern similar to the full-length form after both treatments. On the other hand, the co-IP with E70 was enriched in the unglycosylated forms, as indicated by the limited extent of changes induced by enzymatic treatment (Fig. 4*A*), suggesting that E70 interacts preferentially with the nonglycosylated full-length channel. When different ratios of full-length K_V_10.1 and E70 were injected into *Xenopus* oocytes, the fraction of the complex glycosylated full-length version decreased with increasing amount of E70, whereas the abundance of E110 increased concomitantly (Fig. 4, *B* and *C*), portending that E70 reduces complete maturation of the full-length protein.

In contrast, E65 did not alter the glycosylation of full-length K_V_10.1, but rather it affected the total amount of full-length expressed in *Xenopus* oocytes co-injected with both K_V_10.1 and E65. The full-length K_V_10.1 band was virtually abolished when co-expressed with E65 at a 1:10 ratio (Fig. 4*D*).

The surface expression of K_V_10.1 was not diminished by co-expression of E65 or E70. Cells expressing K_V_10.1 containing an extracellular bungarotoxin binding site (K_V_10.1-BBS) were transfected with E65 or E70 (in pcDNA3) in HEK cells. The cells were treated with BTX on ice to avoid internalization and bound BTX was determined by ELISA (Fig. 4*E*). None of the splice variants induced a reduction in surface labeling as compared with the control cells (nontransfected or transfected with empty vector).

##### E65 and E70 Down-regulate K_V_10.1 Currents in Xenopus laevis Oocytes and HEK293 Cells

**FIGURE 5.**
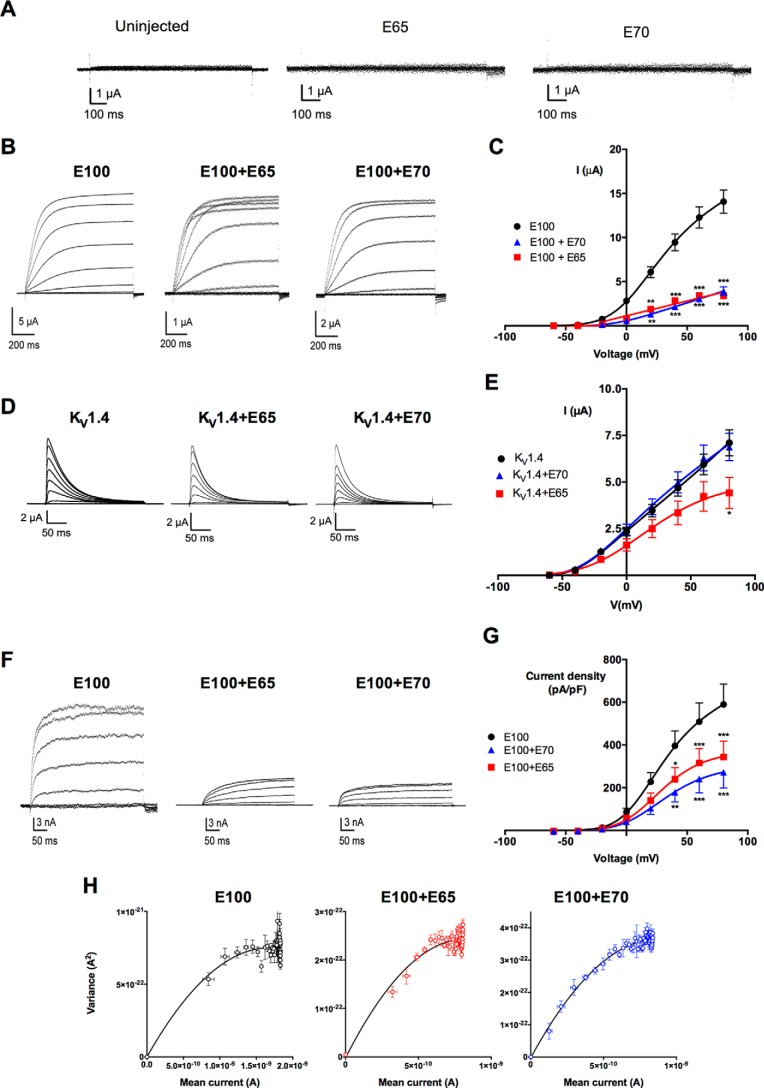
**K_V_10.1 current is down-regulated on co-expression with E65 and E70 in two different heterologous expression systems.** *A*, E65 or E70 alone fails to generate potassium currents in oocytes. Traces were obtained from oocytes injected with E70, E65, or uninjected. None of the conditions gave rise to significant potassium currents. *B*, representative current traces for full-length K_V_10.1 (E100, *left panel*), E100 + E65 (*middle panel*), and E100 + E70 (*right panel*) from voltage clamp experiments in *Xenopus* oocytes. *C*, current-voltage relationship for E100 (*black circles*, *n* = 20), E100 + E65 (*red squares*, *n* = 16), and E100 + E70 (*blue triangles*, *n* = 20). *D*, current traces obtained from oocytes expressing K_V_1.4 either alone or together with E65 or E70. Current kinetics was not altered by co-expression. *E*, current-voltage relationship in oocytes expressing K_V_1.4 together with E65 or E70 1:10. E70 had no detectable effects on the current (*n* = 24), whereas E65 induced a modest reduction (37 ± 16%, *p* = 0.02 at +80 mV, *n* = 18). The effects of E65 alone on the properties of the oocytes could be responsible in part for this phenomenon. *F*, representative traces from patch clamp experiments with HEK cells stably expressing E100 and transfected with mVenus (*left panel*), E65mVenus (*middle panel*), or E70mVenus (*right panel*). Only green fluorescent cells visualized under an epifluorescence microscopy were used for recordings. *G*, current-voltage relationship for HEK-K_V_10.1 (*black circles*, E100, *n* = 12), E100 + E70 (*blue triangles*, *n* = 11), and E100 + E65 (*red squares*, *n* = 13). *, *p* < 0.05; **, *p* < 0.01; ***, *p* < 0.001. *H*, representative plots of variance *versus* mean current for K_V_10.1 (*black*), K_V_10.1 + E65 (*red*), and K_V_10.1 + E70 (*blue*), with both E65 and E70 injected at a 1:10 ratio. The parameters resulting in the fit shown are detailed in “Results.”

The interaction of K_V_10.1 with short isoforms also had a functional impact. When cRNAs encoding for E65 or E70 were injected into *Xenopus* oocytes, the current measured in these oocytes with two-electrode voltage clamp was indistinguishable from that measured in uninjected oocytes (Fig. 5*A*). This was expected because both short forms lack the transmembrane segments and should therefore not form functional channels. Co-injection of either E65 or E70 with K_V_10.1 cRNA at a 1:10 ratio resulted in a strong reduction (85 and 60%, respectively) of the current (Fig. 5, *B* and *C*). Furthermore, the presence of E65 induced an intense inward rectification; depolarizations to a very positive potential induced current amplitudes that were smaller than those induced by less intense stimuli (Fig. 5*B*, *middle trace*). Neither E65 nor E70 expression influenced massively the appearance or amplitude of current elicited by another voltage-gated potassium channel (K_V_1.4) (Fig. 5, *D* and *E*), indicating that the interaction with E65 or E70 is specific for K_V_10.1. Although E65 induced a modest reduction (37 ± 16%, *p* = 0.02 at +80 mV, *n* = 18) in the current of K_V_1.4, this effect arguably could be explained by the modification that E65 alone induces on the oocyte itself (see below).

The reduction in current amplitude observed in oocytes is unlikely to be due to the specific expression system, because comparable results were obtained from HEK293 cells stably expressing K_V_10.1 and transiently co-transfected with E65mVenus or E70mVenus (using mVenus alone as a control) and measured 24–72 h after transfection in whole-cell patch clamp experiments (Fig. 5, *F* and *G*). mVenus fluorescence was used to select transfected cells for recording. E65 and E70 expression resulted in a marked K_V_10.1 current reduction of 41.7 ± 20.5% for E65 and 53.8 ± 21.1% for E70 (*p* < 0.0001, two-way analysis of variance (Fig. 5*G*)).

A reduction in the total current measured can be the result of a smaller current through each open channel, a reduced open probability, or a smaller number of available channels, which are not necessarily all that are present on the membrane (whose abundance is not changed or only little changed (Fig. 4*E*)). To distinguish between these possibilities, we performed nonstationary noise analysis in membrane patches from oocytes expressing E100 either alone or in combination (at 1:10 ratio) with E65 or E70. As summarized in Fig. 5*H*, neither of the isoforms reduced the open probability at +40 mV (0.48 ± 0.17, 0.46 ± 0.13, and 0.42 ± 0.15 for E100, E100 + E65, and E100 + E70, respectively). Neither did E70 change the single channel current (886 ± 85 fA for E100 *versus* 829 ± 37 fA for E100 + E70), indicating that the number of available channels is the factor that is reduced in this combination, despite the fact that the total channels on the membrane do not seem to change. In contrast, E65 did induce a reduction (to 593 ± 42 fA), again pointing to a mechanistically different action of the two variants.

##### Role of the Tetramerization Domain in the Physical and Functional Interaction between E65 and E70 and the Full-length K_V_10.1

**FIGURE 6.**
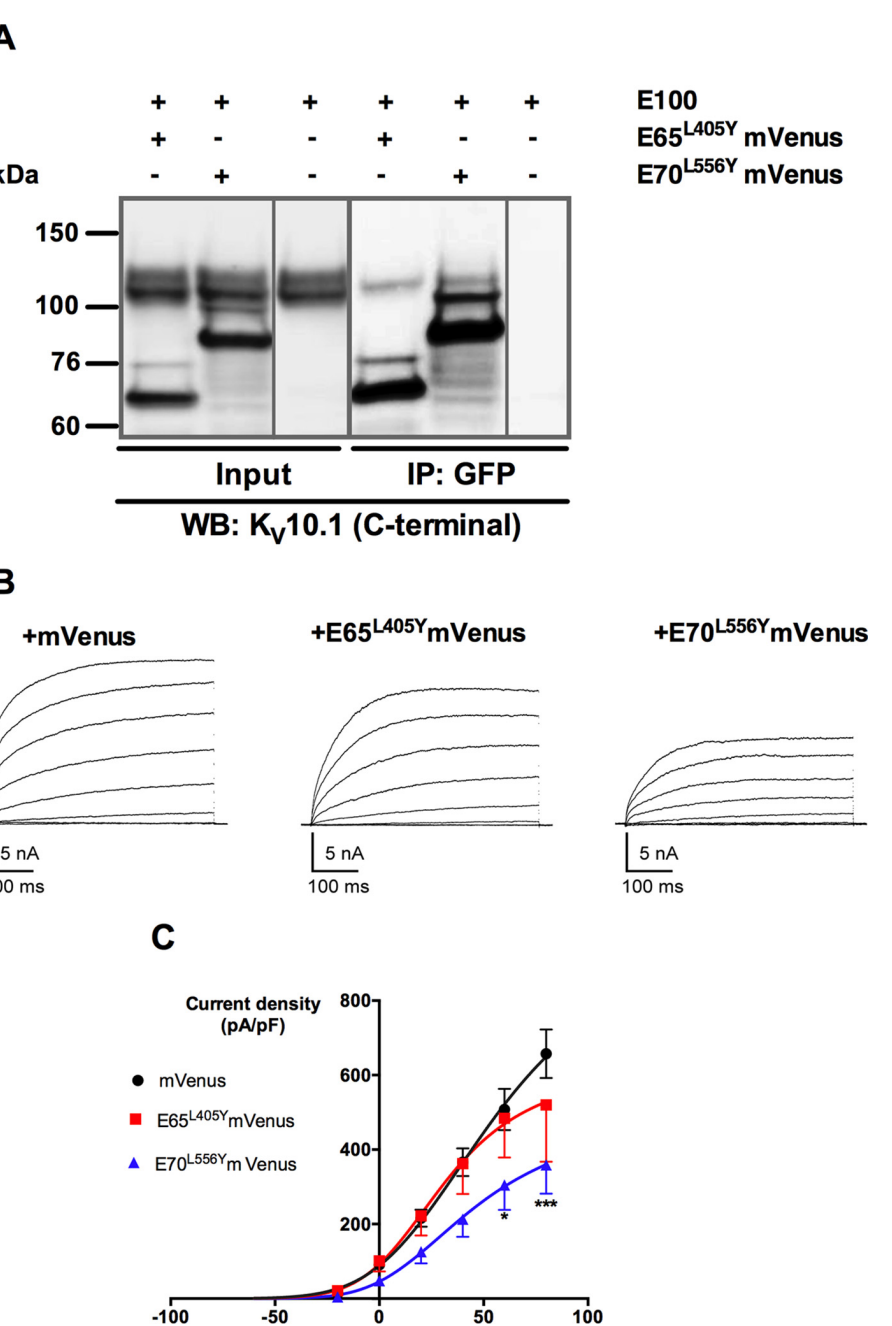
**TCC is not necessary for the interaction between K_V_10.1 and the splice variant E70 but impairs the current reduction induced by E65.** *A*, immunoprecipitation of mVenus-fused splice variants E65^L405Y^ and E70^L556Y^ transiently expressed in HEK cells with anti-GFP antibody co-precipitated K_V_10.1 protein (∼130 kDa). *Input*, 10% (20 μg) of cell extracts. *B*, patch clamp recordings of HEK cells, with typical current traces from transfected cells co-expressing mVenus (*left panel*), E100 + E65^L405Y^mVenus (*middle panel*), or E100+E70^L556Y^mVenus (*right panel*). Only green fluorescent cells visualized under an epifluorescence microscope were used for recordings. *C*, *I*-V graph representing the mean current density at each voltage step for HEK- K_V_10.1 transfected with mVenus (*black circles*), E65^L405Y^mVenus (*red squares*), and E70^L556Y^mVenus (*blue triangles*). *, *p* < 0.05; ***, *p* < 0.001).

The TCC domain is a 41-amino acid sequence in the C terminus of K_V_10.1 that is important for specific and stable tetrameric assembly. A point mutation at position 20 within this sequence (L913Y) disrupts the coiled-coil domain and tetramerization (19). Here, this mutation was used to test the requirement of the TCC motif for the interaction between short isoforms and full-length K_V_10.1. E65 and E70 mutated at the corresponding position co-precipitated with K_V_10.1, although in E65^L405Y^ co-IP was less efficient than in E70^L556Y^ (Fig. 6*A*). Therefore, we determined that an intact TCC is not required for the physical interaction between isoforms.

K_V_10.1 functional properties were also determined when co-expressed with the mutant short variants in whole-cell patch clamp experiments (Fig. 6, *B* and *C*). E70^L556Y^ induced a reduction in the K_V_10.1 current density by 45.3 ± 20.3% (*p* < 0.001, two-way analysis of variance) at +80 mV (Fig. 6*C*), an effect similar to wild type E70. In contrast, E65^L405Y^ reduced the current amplitude only slightly, and the difference did not reach statistical significance (20.8 ± 21.9% (Fig. 6*C*)). These results strongly suggest that although TCC is not required for the interaction, it plays a critical role in the E65-induced K_V_10.1 current decrease.

##### E65 Expression Triggers Cell Cycle Progression in Xenopus oocytes

**FIGURE 7.**
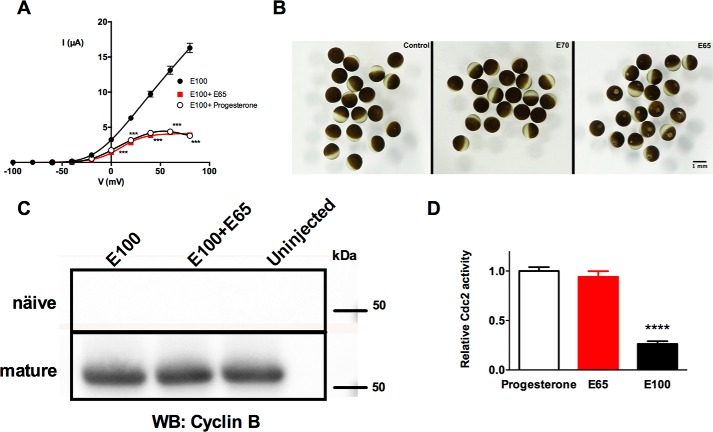
**Expression of E65 in *Xenopus* oocytes induces oocyte maturation independently of cyclin B and increases Cdc2 kinase activity.** *A*, *I*-V relationship corresponding to the current for E100 (*black*), E100 + E65 in a 1:10 ratio (*red*), and E100 + progesterone pulse (*open circles*). ***, *p* < 0.001; *asterisks* apply for the full-length K_V_10.1 + E65 and also the progesterone pulse. *B*, GVBD was visible in oocytes injected with RNase-treated extract from E65-expressing oocytes (*right panel*) but not with RNase-treated extract from uninjected oocytes (*left panel*) or injected with extract from E70-injected oocytes (*middle panel*). *C*, extracts from naive (*upper panel*) or mature (progesterone-treated, *lower panel*) oocytes (*n* = 15) injected with the indicated cRNA combinations were immunoprecipitated and immunoblotted using an anti-cyclin B2 monoclonal antibody. *D*, Cdc2/Cdk1 activity was determined by ELISA under the described conditions. ****, *p* < 0.0001.

*X. laevis* oocytes are physiologically arrested in the late G_2_ phase of the first meiotic division. The application of progesterone triggers activation of a mitosis promoting factor (cyclin B/p34^Cdc2^) and progression to meiosis II in a process called maturation. When K_V_10.1 channels are expressed in *Xenopus* oocytes, the maturation of the oocyte induces a reduction in current amplitude and a strong inward rectification due to a block by intracellular sodium (60, 61). Here we found that the current phenotype resulting from recordings of oocytes co-injected with full-length K_V_10.1 and E65 is very similar to that observed in mature oocytes (Fig. 7*A*). Such a phenotype was detected despite the fact that the physiological maturation of the oocytes was inhibited by using theophylline in the oocyte medium. We therefore hypothesized that E65 itself could induce maturation of the oocyte. Active Cdc2 present in mature oocytes can trigger the cascade when a cytoplasmic extract of mature oocytes is injected in immature oocytes. When an extract from E65-expressing oocytes was injected into naive oocytes (followed by RNase treatment to eliminate the preinjected cRNA), germinal vesicle breakdown (GVBD), the hallmark of maturation, was detected 2 h post-injection (Fig. 7*B* and Table 3). Injection of RNase-treated extracts of control oocytes or of extracts from oocytes injected with E70 did not induce GVBD.

**TABLE 3:** **Germinal vesicle breakdown**

Treatment	Injected oocytes	Visible GVBD	Not evaluable
E65 extract	109	64	13
E70 extract	20		
Uninjected extract	80		10
K_V_10.1 cRNA	30		

An early and decisive event in the process of the physiological maturation of the oocyte is an increase in the abundance of cyclin B2. The action of E65 appears to happen downstream of this step, because induction of maturation by E65 did not require an increase in the levels of cyclin B2 (Fig. 7*C*). Indeed, cyclin B2 levels were unaffected in E65-expressing and naive oocytes compared with uninjected and E100-injected samples, both before and after the physiological induction of maturation with progesterone (5 μg/ml) (Fig. 7*C*). Nevertheless, we could detect high Cdc2 kinase activity in E65-expressing oocytes, comparable to the activity observed in the progesterone-treated samples (Fig. 7*D*). This could explain the induction of GVBD. Oocytes injected with E100 showed a lower Cdc2 kinase activity, and in uninjected oocytes, we did not observe Cdc2 activity (not showed). We concluded that E65 induces maturation of the oocyte by direct activation of Cdc2 without an increase in cyclin B levels. However, we did not observe a consistent and reproducible change in cell cycle distribution in mammalian cells expressing K_V_10.1 upon overexpression of E65 or E70 (data not shown).

## Discussion

We have isolated two novel splice variants of K_V_10.1. Alternative splicing can induce profound modifications in the properties of proteins, including ion channels. We hypothesized that the differences between properties of the human melanoma cell lines IGR39 and IPC298 could be due to alternative splicing. IGR39 melanoma cells express K_V_10.1 at the transcript and protein levels. However, no current with kinetics suggestive of this channel was detected, although the pharmacology of the current in IGR39 cells was similar to that of K_V_10.1. Most importantly, the current was abolished by K_V_10.1-specific siRNA (Fig. 1*F*). Based on the immunocytochemistry shown in Fig. 1, our initial hypothesis was the presence of an alternative isoform containing the K_V_10.1 pore but not the C-terminal region (Fig. 1*C*). However, we were not able to isolate such a variant but rather a novel short variant (E65) that does contain a canonical C terminus. It still needs to be unequivocally clarified why the C-terminal region in IGR39 cells is detected in immunoprecipitation but not in immunocytochemistry experiments, but a possible explanation is that the epitope for mAb33 is three-dimensional.^6^ The human brain expresses a different short isoform (E70), which is also detected in neuroblastoma cell lines and IPC298 melanoma cells.

None of the splice variants could be detected as protein in native systems, and therefore we assumed scarce expression, either because their expression is actually low or because they are present only in a subset of cells. The short variants may represent orthologs of the known Eag80 splice variants described in *Drosophila* (49).

The predicted products of E65 and E70 would bear all of the functional domains described for the C terminus but would lack all transmembrane segments and part of the PAS domain. The cyclic nucleotide binding domain and the PAS domain have been described as interacting with and modulating channel gating (62); the interacting residues are still present in the splice variants, and it is therefore conceivable that the interactions still exist.

Several non-channel proteins resulting from alternative splicing events have been described as modulating the biophysical properties of the corresponding full-length channel (37, 41, 44–46). Both E65 and E70 interact with the full-length K_V_10.1, leading to variant-specific biological consequences. E65 causes an overall reduction in K_V_10.1 protein level (Fig. 4*D*) and reduces single channel current, whereas E70 diminishes the ratio of complex-to-core glycosylated K_V_10.1 in a dose-dependent manner (Fig. 4, *B* and *C*) without detectably affecting the electrophysiological parameters.

The molecular interaction between the short isoforms and the full-length K_V_10.1 could involve heterotetramerization between the isoforms that would render the channel inactive (63), as all four pore loops are required to generate an active channel. Both E65 and E70 retain the TCC domain. However, we found that TCC-defective E65 and E70 short isoforms conserve the interaction with K_V_10.1 (Fig. 6*A*). Consequently, the interaction between the short isoforms and the full-length channel would occur through other interaction site(s).

Disruption of the coiled structure of the TCC abolished the reduction in current induced by E65, but E70 retained the ability to diminish the current. This is consistent with an action of E65 involving interference with K^+^ permeation (thereby reducing conductance), whereas E70 rather reduces the amount of channels that can be activated, although the overall abundance of membrane channels is not affected by either isoform. Lack of proper glycosylation is known to render inactive channels, even if this happens after the channels have been inserted in the plasma membrane (29). Therefore, an impairment of K_V_101 glycosylation would be enough to explain the observations with E70.

The effect of E65 requires a more complex interpretation. Besides a strongly reduced amount of K_V_10.1 current, *X. laevis* oocytes co-expressing K_V_10.1 and E65 show a dramatic voltage-dependent inward rectification that can be explained because E65 induces the activation of Cdc2 independently of cyclin B (Fig. 7, *C* and *D*). Injection of the full-length channel alone also induces a significant increase in Cdc2 activity, which could be due to the processing of E100 RNA to produce E65 (49) although we have not tested this hypothesis further. Such an effect would potentially affect the measurements depicted in Fig. 5*H*. Although rectification in mature oocytes is a phenomenon attributable in this case to block by intracellular sodium (61), and this cation was not present in the internal solution, the reduction in the single channel current could be due to the maturation of the oocyte rather than a direct action of E65 on the channel. The effects on cdc2 activity, although a potentially interesting observation with pathophysiological consequences, fall outside the focus of the present study and therefore were not further explored.

Taken together, our results highlight the complexity of action K_V_10.1 in the context of tumorigenesis, which can be cell type (and therefore tumor)-specific. They also underline the relevance of the noncanonical effects of ion channels in both physiological (as in the case of E70 in the brain) and pathological (for example, E65) conditions.

## Author Contributions

L. A. P. designed the research; C. W. initiated the project; and F. R. G., V. R., A. S., C. W., P. N., M. P., and L. A. P. designed, performed, and analyzed the experiments. F. R. G., V. R., A. S., C. W., and L. A. P. participated in writing the manuscript.

## Footnotes

6 L. A. Pardo, unpublished observations.

## Footnotes

5 The abbreviations used are:

TCC: tetramerizing coiled-coil
BTX: bungarotoxin
ABTS: 2,2′-azino-bis(3-ethylbenzothiazoline-6-sulfonic acid)
co-IP: co-immunoprecipitation
GVBD: germinal vesicle breakdown.
